# Skin-Whitening Effect of a Callus Extract of *Nelumbo nucifera* Isolate Haman

**DOI:** 10.3390/plants12233923

**Published:** 2023-11-21

**Authors:** Sung Ho Moon, Euihyun Kim, Hye-In Kim, Soo-Yun Kim, Hyo-Hyun Seo, Jeong Hun Lee, Min-Sup Lee, Seung-Ki Lee, Sang Hyun Moh, Seunghee Bae

**Affiliations:** 1 Department of Cosmetics Engineering, Konkuk University, Seoul 05029, Republic of Korea; shmoon1999@konkuk.ac.kr; 2 Plant Cell Research Institute of BIO-FD&C Co., Ltd., Incheon 21990, Republic of Korea; hyun9214@snu.ac.kr (E.K.); hikim@biofdnc.com (H.-I.K.); sykim@biofdnc.com (S.-Y.K.); hhseo@biofdnc.com (H.-H.S.); jhlee@biofdnc.com (J.H.L.); 3 Biotechnology Research Institute, Celltrion, Inc., Incheon 22014, Republic of Korea; minsup.lee@celltrion.com (M.-S.L.); seungki.lee@celltrion.com (S.-K.L.)

**Keywords:** lotus, *Nelumbo nucifera*, whitening, extract, skin, callus

## Abstract

The sacred lotus (*Nelumbo nucifera* Gaertn. Isolate Haman, in the family Nelumbonaceae) used in this study originated from the Haman region of Korea, and lotus seeds dating back to the Goryeo Dynasty (650–760 years ago) were accidentally discovered. Lotus is known to possess antioxidant, anti-inflammatory, and soothing properties. Instead of using the lotus alone, we obtained extracts using Haman region lotus-derived callus (HLC), which allowed for a controlled, quantitative, and infinite supply. Based on the reported effects of the lotus, we formulated a hypothesis to investigate the skin-whitening effect of the HLC extract (HLCE). The HLCE was first obtained by extraction with distilled water and using 5% propanediol as a solvent and subsequently verified for the whitening effect (melanin content tests) using mammalian cells in vitro. Its efficacy at the molecular level was confirmed through real-time polymerase chain reaction (PCR) using melanin-related genes. Furthermore, clinical trials with 21 volunteers confirmed the significant whitening effect of cosmetics containing the HLCE. In conclusion, we found that the HLCE not only has anti-inflammatory, antioxidant, and skin-soothing properties but also plays an essential role in skin whitening. Therefore, we propose that the HLCE has the potential to become a new raw material for the cosmetic industry.

## 1. Introduction

**Figure 1:**
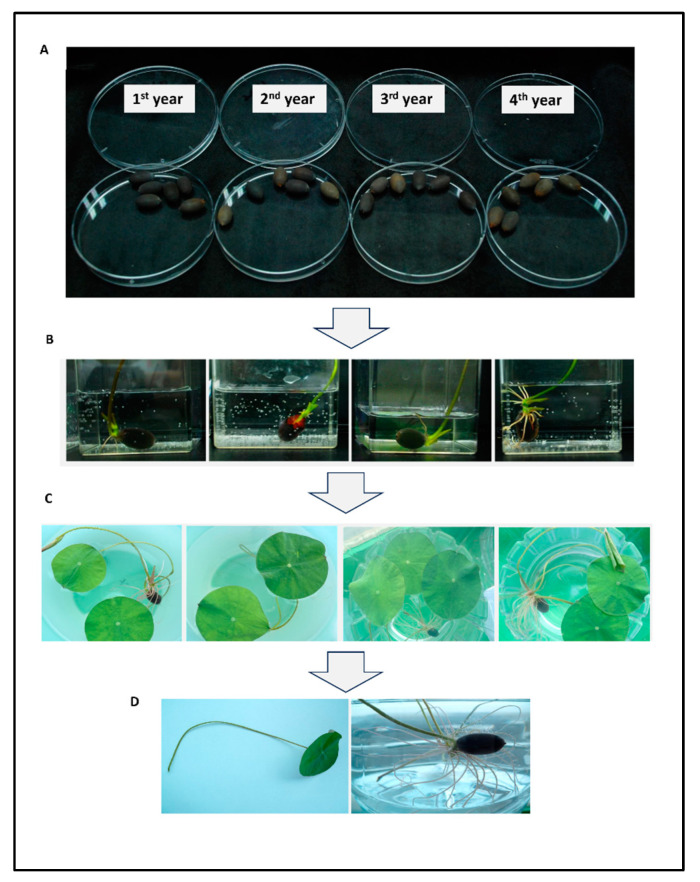
Procedure of Haman region lotus seed germination. (**A**) Annually collected seeds from Haman, Korea. (**B**) The seeds were cultured in water until the germination and (**C**,**D**) the seed sprouts were cultured to obtain the first leaves. Subsequently, the leaves were obtained and utilized for callus induction.

Sacred lotus (*Nelumbo nucifera* Gaertn. isolate Haman in the family Nelumbonaceae) is a perennial herb that lives in the southern part of Asia, northern Australia, and Russia. The flowers, seeds, leaves, and roots of lotus plants have continuously been used for medicine and food; moreover, they are known to have numerous functions such as self-cleaning [1], anti-ischemia [2], antioxidant [3,4,5], anti-inflammatory [6], and hypocholesterolemic functions [7], and the effects currently have been applicated to synthesis technologies, such as waterproofing in window glass and solar cell panels [8,9]. Likewise, there have already been numerous discoveries of the efficacies of the lotus. Shen-Miller et al. once reported that a 1300-year-old seed from the sacred lotus (*Nelumbo nucifera*) fruit was found to be the oldest seed that had ever been germinated [10]. Similarly, in May 2009, during the excavation of the Haman Sungsan Mountain fortress, three ancient lotus seeds were found. According to the results of the Korea Institute of Geoscience and Mineral Resources, it was confirmed that it was from the Goryeo period from 650 to 760 years ago by radiocarbon dating using ^14^C. One of the three seeds was successfully germinated and, subsequently, 30 seeds harvested for three years from 2010 to 2012 were obtained from the Haman Museum in Haman-gun, Gyeongsangnam-do (Figure 1).

To date, studies in the field of cosmetics have reported the effects of extracts obtained from plants or dried plants on human skin cells [11,12,13,14]. However, there has been very little validation of the effects of extracts from the plant callus, which differ from those of whole plants. Calluses are typically cultured for purposes such as dedifferentiation, gene editing, or plasmolysis [15,16,17]; through these experiments, calluses have been extensively studied as a lab-scale plant model. Several studies have been conducted on the callus extract [18,19]. Occasionally, they are published in domestic journals or registered in domestic patents; however, there are few precise research papers documenting these studies or applications in the cosmetics industry. The advantages of callus, such as sterility, infinite supply, and the potential to obtain extracts from them with highly accurate yields, are worthy of consideration.

The whitening effects of cosmetics have evolved since ancient Greece [20] and various raw materials with skin-whitening effects have been discovered [21]. In particular, the human skin is divided into the epidermis, which has a thickness of 50 to 100 µm, and the dermis, which has a thickness of 2–3 mm. The epidermis is further divided into the stratum corneum and the granular, spinous, and basal layers [22]. Among them, there are melanocytes, which are cells that produce melanin, in the basal layer. When these cells are stimulated by high levels of ultraviolet radiation, tyrosinase oxidizes tyrosine in melanocytes. Therefore, oxidized tyrosine produces melanin, which accumulates, and cells containing melanin take approximately 30 d to reach the stratum corneum [23]. Numerous studies investigating this mechanism have been published [24,25,26].

Several studies have reported about the whitening effects of lotus extracts. Juhasz et al. commented that lotus extract could be applied for whitening, as it was proven that the extract affected melanoma cell death, photoprotection, and wound healing, which would support skin whitening [27]. Moreover, Kim et al. demonstrated that lotus seeds improved the whitening and anti-wrinkle effects through 3,4-dihydroxyphenylalanin-oxidase inhibition and tyrosinase (TYR) inhibition assays [28]. These results suggest that the lotus has significant potential for use in skin cosmetics. Based on the above findings, we assumed that Haman region lotus-derived callus extract (HLCE) would have similar properties to the lotus extract. Therefore, the main purpose of this study was to examine the skin-whitening effects of HLCE using several in vitro, gene expression, and clinical tests. 

## 2. Results

### 2.1. Seed Germination, Callus Induction, Cell Culture, Extraction

**Figure 2:**
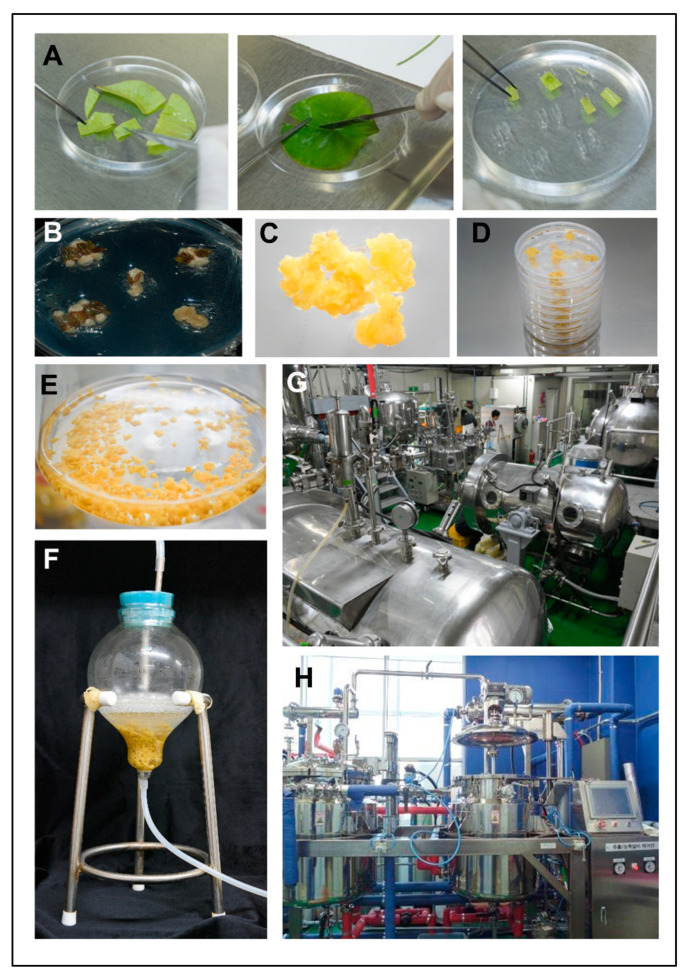
Procedure of Haman region lotus callus induction and callus culture. (**A**) Incising the first leaves of the lotus for plating on the callus induction medium. (**B**–**D**) The calluses were induced within several weeks and selected depending on the color and morphology. Subsequently, when a sufficient amount of callus is obtained, the culture system is scaled up starting from (**E**) agitation culture in a liquid medium, (**F**) bioreactor culture, and (**G**,**H**) mass-scale culture. The callus from the mass-scale culture were not used in this study.

**Figure 3:**
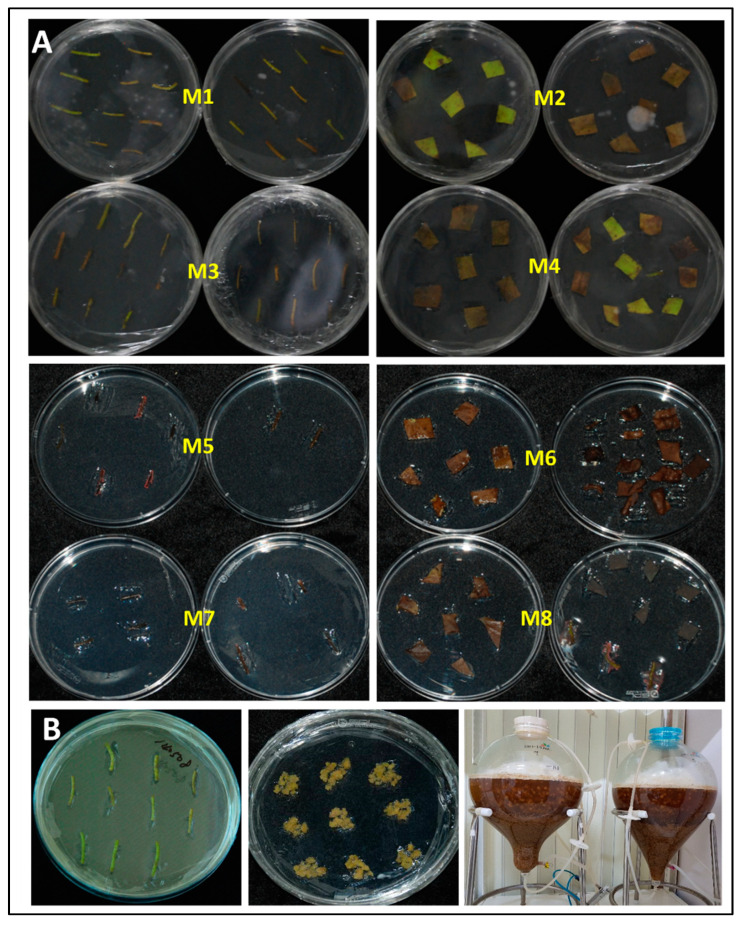
Testing of the callus induction medium. (**A**) The leaves, including the hypocotyls of the lotus, were incised and mounted on the induction media to select the optimal medium for callus growth. (**B**) A brief figure showing callus induction from the hypocotyl of the lotus. M: medium.

Owing to the limited number of seeds provided from the Haman region, we attempted to germinate the seeds obtained every year (first to fourth year; Figure 1A) and succeeded in germinating the seeds by soaking them in water for several days or weeks to induce HLC (Figure 1B). After germination, the plantlets were cultured until the first leaves was observed (Figure 1C,D), sampled, cut into uniform sizes (Figure 2A), and tested in various types of callus-induction media (Figure 2B and Figure 3A) to identify the medium that showed optimal callus induction, cell growth, and cell proliferation. The compositions of the selected media are listed in Table 1 and other media compositions are shown in the supplementary material. We found that the culture medium named ‘M4′ showed the most optimal callus condition, having a bright yellow color (Figure 2C). Subsequently, we successfully cultured HLC from the plate in a 0.5-ton bioreactor (Figure 2D–H). In addition, callus was induced from the lotus hypocotyl and cultured in up to 10 L bioreactors (Figure 3B). Extracts from both first leaf- and hypocotyl-derived callus were tested in vitro for their efficacy. The leaf extract was more effective than the hypocotyl extract. Therefore, the HLCE from leaf was selected for subsequent experiments.

### 2.2. Measurement of Cell Viability

**Figure 4:**
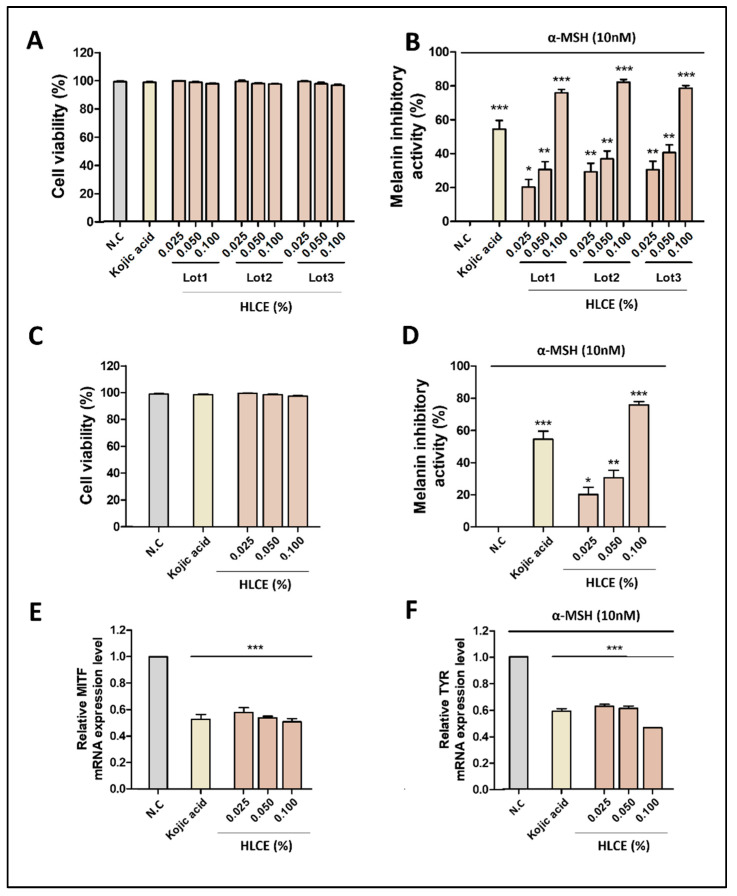
Effects of the HLCE on cell viability and melanin inhibitory activity. (**A**,**B**) A series of batches of HLCE was primarily tested on cells to evaluate cell viability and melanin inhibitory activity, (**C**,**D**) then a significant batch was randomly chosen according to the results from the previous data, proceeded to the same test, and utilized for subsequent experiments. (**E**,**F**) Analysis of mRNA expressions related to skin whitening. α-MSH: Melanin stimulating hormone; N.C: Negative control. *: *p* < 0.05; **: *p* < 0.01; ***: *p* < 0.001.

The cell viability of cultured B16F1 melanoma cells was measured using the 3-(4,5-dimethylthiazol-2-yl)-2,5-diphenyltetrazolium bromide (MTT) assay. Since there are no reports on HLCE treatment at the optimal concentration, serial dilutions of the HLCE ranging from the control, 0.025, 0.05, and 0.1% were added to the cells following our production manual. The extract was obtained thrice to verify the experiment and named as Lot1, Lot2, and Lot3. None of the concentrations showed toxicity compared to the control and positive control (P.C) (Figure 4A). Subsequently, concentrations were randomly selected from different lots and showed no toxicity compared to the control and P.C (Figure 4C). Therefore, we found that HLCE was non-toxic to the cells, indicating that at least the extract itself does not have any primary harmful effects on the cells, and these concentrations were utilized for subsequent experiments.

### 2.3. Measurement of Melanin Content

The whitening effect of cultured B16F1 melanoma cells was confirmed using a melanin synthesis inhibition test. The test results were compared to the α-melanin stimulating hormone (MSH) (10 nM) treated control group (negative control). The values for each sample were averaged and statistical analyses were performed to confirm significance. As shown in Figure 4B,D, the HLCE concentrate showed a noticeable decrease in melanin synthesis in melanoma cells. The test results showed that the treatment of the HLCE concentrate at 0.025–0.1% had a significant melanin synthesis inhibitory effect. At an HLCE concentrate concentration of 0.025%, each lot sample showed a significant inhibition rate of melanin synthesis of the HLCE (Lot1; =20.26%), HLCE (Lot2; 29.17%), and HLCE (Lot3; 30.52%). In addition, at 0.1%, the test results exhibited the melanin synthesis inhibitory effect of HLCE (Lot1; 75.80%), HLCE (Lot2; 82.18%), and HLCE (Lot3; 78.71%) (Figure 4B). Moreover, each concentration selected randomly from different lots showed similar effects (Figure 4D). The cellular melanin synthesis inhibition activity of the HLCE concentrate was approximately 26.65% at 0.025%, 36.02% at 0.050%, and 78.89% at 0.100%, on average. The inhibitory effect on melanin synthesis was dose dependent. Kojic acid, used as P.C, showed a suppression rate of 54.52% at 200 ppm. These results improved the reliability and accuracy of the test by being similar to those of the usual melanin test. In conclusion, the HLCE concentrate exhibited a significant whitening effect at the cellular level.

### 2.4. Quantitative Real-Time Polymerase Chain Reaction (PCR)

To evaluate the effects of HLCE on melanogenesis at the molecular level, we measured the expression of key melanogenic genes, including *Microphthalmia-associated transcription factor* (*MITF*) and *TYR* in B16F1 melanoma cells using quantitative reverse transcription (qRT)-PCR. *MITF* is considered the master regulator of pigmentation and transcriptionally activates melanogenic enzymes such as *TYR*. As shown in Figure 4E,F, the relative expression levels of *MITF* and *TYR* mRNAs were strongly suppressed after HLCE treatment compared to the α-MSH-treated control group (N.C). The results showed that the treatment of HLCE at 0.025–0.1% led to the significant inhibition of the mRNA expression of *MITF* and *TYR* in a dose-dependent manner. As expected, kojic acid (P.C) decreased the relative expression levels of *MITF* and *TYR* mRNAs. The effect of HLCE on melanogenesis-related gene downregulation suggests a possible functional role of HLCE in skin whitening.

### 2.5. Clinical Tests

**Figure 5:**
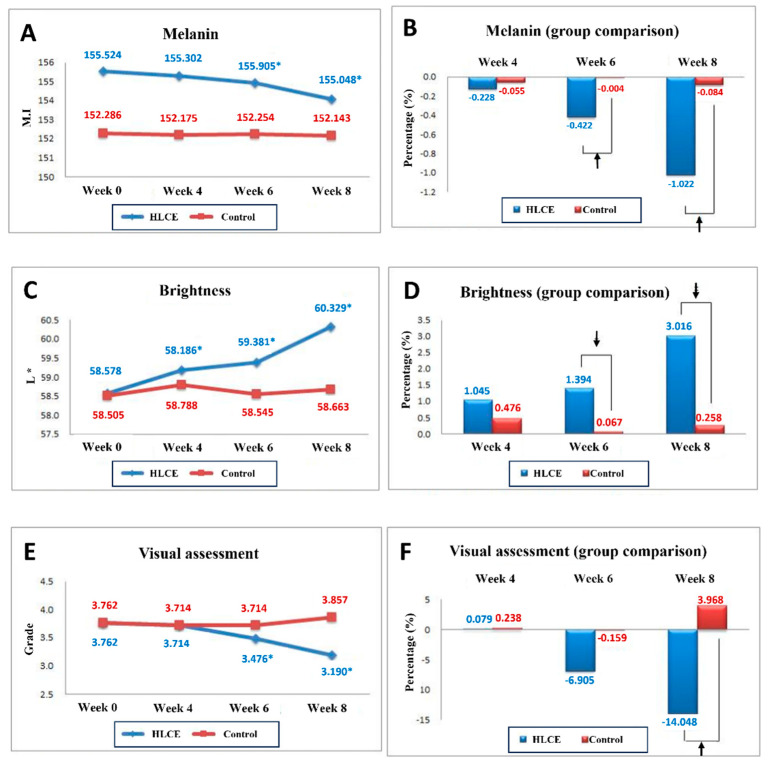
Results from the clinical test of the HLCE cream. (**A**,**B**) The melanin content was measured and expressed as the melanin inhibition index (MI) and percentage. (**C**,**D**) Subsequently, the effect of the HLCE cream was evaluated by skin brightness and (**E**,**F**) the visual assessment was evaluated by clinicians. (**A**,**C**,**E**): Asterisks indicate *p* < 0.05 (0.017) (=5%/3) by repeated measures ANOVA, post hoc paired t-test with Bonferroni correction; (**B**,**D**,**F**): rate of change (%) = (after-before)/before × 100 (Average value of individual rates of change, arrows indicate a statistically significant difference between the two groups (*p* < 0.05) by Mann–Whitney U test); arrows indicate *p* < 0.05 (0.017) (=5%/3) by Friedman test, post hoc Wilcoxon signed rank test with Bonferroni correction.

**Figure 6:**
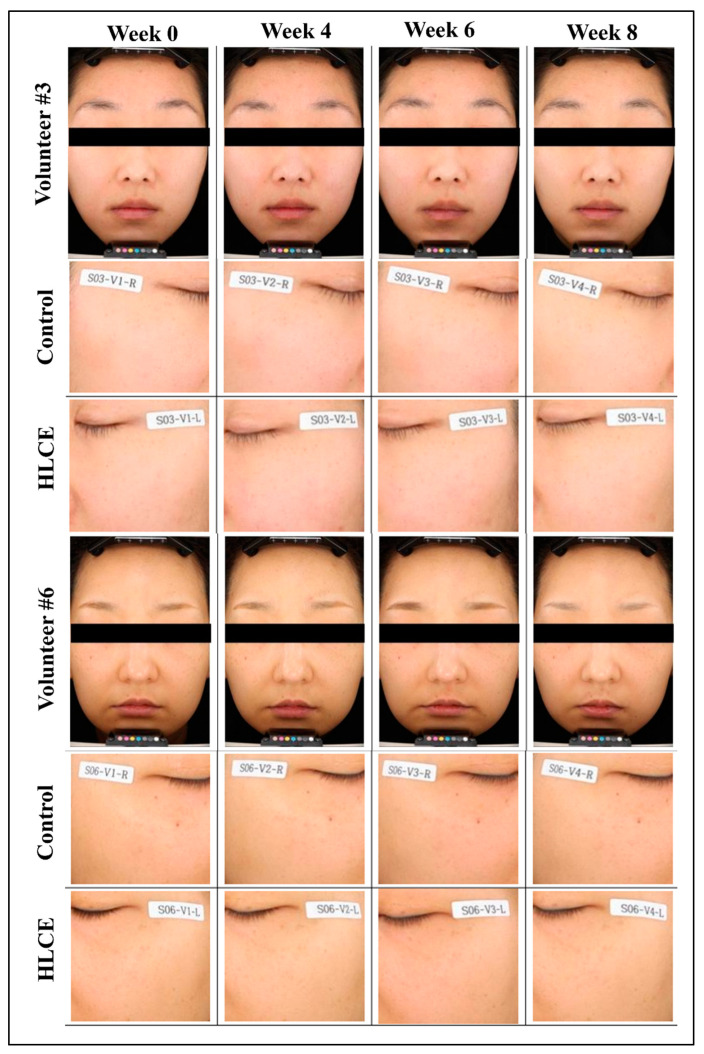
Exemplary figure of the volunteers who participated in the HLCE clinical test. Among the 21 female participants, volunteers 3 and 6 showed the highest melanin inhibition values. The effect was visible starting from the sixth week of administration.

(1) Safety evaluation: During the clinical trial, none of the subjects reported experiencing any negative skin responses, and the dermatologist’s physical examination did not reveal any unusual observations.

(2) Skin melanin (Mexameter): As shown in Figure 5A,B, skin melanin levels were evaluated initially and at 4, 6, and 8 weeks to confirm any changes induced by the tested product. In the placebo group, the initial skin melanin measurement was 152.286 ± 25.242, which changed to 152.175 ± 25.052 after 4 weeks of product use, 152.254 ± 25.099 after 6 weeks, and 152.143 ± 25.115 after 8 weeks. For the group using the cream containing HLCE, the initial skin melanin measurement was 155.524 ± 38.186 and decreased to 155.302 ± 38.101 after 4 weeks of product use, 154.905 ± 38.213 after 6 weeks, and 154.048 ± 38.311 after 8 weeks. Notably, the tested product demonstrated a significant reduction in skin melanin levels after 6 and 8 weeks of use compared with the initial measurement (*p* < 0.05). Additionally, there was a statistically significant difference in skin melanin levels between the test and placebo groups after 6 and 8 weeks of treatment (*p* < 0.05).

(3) Skin brightness (CM-700d Spectrophotometer): Skin brightness was assessed at the outset and subsequently at 4, 6, and e8 weeks to confirm alterations in skin brightness resulting from the application of the test product (Figure 5C,D). In the placebo group, the initial measurement of skin brightness was recorded at 58.505 ± 2.431, which then increased to 58.788 ± 2.588 after 4 weeks of product usage, followed by 58.545 ± 2.491 after 6 weeks, and 58.663 ± 2.678 after 8 weeks. For the group using the cream containing HLCE, the initial measurement of skin brightness was 58.578 ± 2.878, then it rose to 59.186 ± 2.918 after 4 weeks of product usage, 59.381 ± 2.730 after 6 weeks, and 60.329 ± 2.684 after 8 weeks. The test product exhibited a noteworthy increase in skin brightness after 4, 6, and 8 weeks of use compared to the initial measurement, and this increase was statistically significant (*p* < 0.05). Moreover, there was a statistically significant difference in skin brightness between the test and placebo products after 6 and 8 weeks of use (*p* < 0.05).

(4) Visual assessment (Researcher): As shown in Figure 5E,F, visual assessments were conducted initially and at 4, 6, and 8 weeks to confirm any changes in the visual assessment scores resulting from the use of the test product. In the placebo group, the initial visual assessment score was 3.762 ± 0.539. This score remained at 3.714 ± 0.463 after 4 weeks of product use, stayed at 3.714 ± 0.463 after 6 weeks, and increased to 3.857 ± 0.478 after 8 weeks. In contrast, for the group using the cream containing HLCE, the initial visual assessment score was also 3.762 ± 0.539. However, it decreased to 3.714 ± 0.561 after 4 weeks of product use, further declined to 3.476 ± 0.512 after 6 weeks, and reached 3.190 ± 0.602 after 8 weeks. The test product exhibited a notable reduction in visual assessment scores after 6 and 8 weeks of use compared with the initial assessment, and this reduction was statistically significant (*p* < 0.05). Additionally, there was a statistically significant difference in the visual assessment scores between the test and placebo products after 8 weeks of use (*p* < 0.05). Visible data from volunteers with whitening effects through HLCE are shown in Figure 6.

## 3. Materials and Methods

### 3.1. Seed Germination, Callus Induction, and Culture

To protect rare species of lotus from being extinguished and facilitate the synthesis and extraction of biologically active substances, stable callus cultures derived from lotus seeds were established. Cell cultures of *N. nucifera* were provided from Haman County. The seeds were sterilized and then grown under water-supplied conditions until small leaves germinated. The leaves were sliced into 1 cm pieces of explants and then transferred to a solid agar plate on Murashige and Skoog (MS) medium with 0.8% agar (*w*/*v*). The medium was supplemented with 30 gL^−1^ sucrose; 0.5 mgL^−1^ α-naphtaleneacetic acid (NAA) and 1 mgL^−1^ 6-benzylaminopurine (BAP). The leaf segments were incubated in the growth chambers at 25 ± 1 °C under dark conditions. After 30 d, the leaf segments began to induce callus formation. The induced calluses were then transferred to a fresh solid MS medium containing 0.3 mgL^−1^ 2,4-dichlorophenoxyacetic acid and 0.8 mgL^−1^ BAP for proliferation. Following two passages (30 d each), calluses that were 0.2~0.3 cm in diameter were used for the development of suspension cultures. Callus suspension cultures were initiated by adding 7 g callus inoculum to a 70 mL MS liquid medium with 30 gL^−1^ sucrose in 250 mL Erlenmeyer flasks. The cultures were kept under continuous agitation at 110 rpm in the orbital shaker and incubated at 25 ± 1 °C. The incubation was carried out for approximately 2 to 3 weeks until the size of the callus reaches a point where it is not agitated by shaking.

### 3.2. Callus Suspension Culture 

The suspension culture was established in 3 L bioreactors along with 2 L of the MS medium supplemented with 0.05 mgL^−1^ NAA, 3.0% (*w*/*v*) sucrose (150 mg L^−1^) to verify the suitable culture method for accumulating biomass and bioactive compounds. Next, 50 gL^−1^ callus (wet mass) was used as the inoculum. The aeration volume in bioreactors was automatically adjusted to 0.1 vvm (air volume per culture volume per minute) using air flow meters (RMA series; Dwyer Instruments Inc., Indiana, MI, USA) and cultures were maintained for 5 weeks at 25 ± 1 °C under dark conditions. Every 4 or 5 weeks, the suspension culture was subcultured and transferred into upper scales (5, 10, and 20 L). Through the 500 L inoculator, 20 L callus cultures were introduced to the mass culture system for 3 to 4 months. Lotus calluses were collected and washed thrice with purified water. The calluses were dried by lyophilization and stored under dark, dry conditions until use. 

### 3.3. Extraction of Lotus Callus Isolate HAMAN

Dried or lyophilized callus was first weighed at 2 g/L and then added to distilled water in an Erlenmeyer flask. The extraction was initiated by placing the flask in a 40 °C water bath for 4 h. Subsequently, the extract was initially filtered through a 40 μm cell strainer (Corning, New York, NY, USA) to remove any callus or other remnants. Then, the extract was filtered again using a 0.22 μm filter (GVS, Bologna, Italy). The resulting extract was then used immediately.

### 3.4. Melanocyte Culture and HLCE Treatment

The highly pigmented mouse skin melanocyte cell line B16F1 was purchased from the American Type Culture Collection. B16F1 cells were cultured in Dulbecco’s modified Eagle’s medium (DMEM) (containing 10% fetal bovine serum; FBS), penicillin (100 U/mL), and streptomycin (100 µg/mL) at 37 °C in a humidified 5% CO_2_ incubator. The cells were subcultured every second day until passage 20. The HLCE used in this study was obtained by extraction with distilled water and using 5% propanediol as the solvent. The extract was added at 0.025, 0.05, and 0.1% concentrations in a serum-free DMEM medium. Kojic acid was used as a P.C at a concentration of 200 ppm. 

### 3.5. Measurement of Cell Viability

The viability of HLCE-treated B16F1 cells was determined using the MTT assay. B16F1 cells were seeded in a 96-well plate at a density of 5 × 10^3^ cells/well and incubated at 37 °C for 24 h. Following overnight incubation, α-MSH (10 nM) was added and cells were treated with various concentrations of the HLCE in defined DMEM for 72 h. Then, 5 μL of MTT (5 mg/mL in PBS) was added to each well and the cells were incubated at 37 °C for 4 h. Subsequently, the medium was gently removed and 100 μL of dimethyl sulfoxide (DMSO) was added to dissolve the formazan crystals. The insoluble purple formazan formed from yellowish MTT by enzymatic reduction was dissolved in DMSO after supernatant removal. The absorbance was measured at 540 nm using a spectrophotometer. Cell viability was calculated using the following equation:

Cell Viability (%) = B/A × 100

A = Absorbance of the control (Shown as ‘N.C’)

B = Absorbance of the sample

### 3.6. Measurement of Melanin Content

The melanin content in B16F1 cells treated with HLCE was determined according to the method described by Hosoi et al., with modifications [29]. B16F1 cells were seeded in a six-well plate at a density of 1 × 10^5^ cells/well for 24 h. Following overnight incubation, α-MSH (10 nM) was added and cells were treated with various concentrations of samples in DMEM for 72 h. Kojic acid (200 ppm), which was considered as the P.C, was used for the assay. The medium was removed and the cells were washed twice with PBS and harvested by trypsinization. The harvested cells were pelleted and solubilized in 400 μL of 1 N NaOH for 30 min at 80 °C. The absorbance of the samples was measured at 405 nm, and melanin concentrations were calculated by comparing the absorbance with a standard curve obtained from synthetic melanin. Each value was normalized to the total protein concentration of each sample using a Bio-Rad protein assay kit (Bio-Rad, Hercules, CA, USA). Untreated cells were used as controls. Melanin production was expressed as the percentage of α-MSH-treated controls. Melanin content was calculated using the following equation:

Melanin inhibitory activity (%) = [(A − B) − (C − B)]/(A − B) × 100

A = Melanin content per unit protein of untreated control

B = Melanin content per unit protein of N.C

C = Melanin content per unit protein of each sample

### 3.7. qRT-PCR

The mRNA expression levels of *MITF* and *TYR* were measured after 72 h in B16F1 cells. Total RNA was isolated from cells using a SuperPrep Cell Lysis kit (TOSCQ-101, TOYOBO), and cDNA was synthesized using a SuperPrep RT kit (TOSCQ-101, TOYOBO). qRT-PCR was performed using the Thunderbird SYBR qPCR Mix (TOQPS-201, TOYOBO) according to the manufacturer’s instructions. Primers for *MITF*, *TYR*, and *GAPDH* were used (QuantiTect^®^ primer assays; QT00037737 for *MITF*, QT00080815 for *TYR*, and QT01192646 for GAPDH, Qiagen). *GAPDH* was used as the control gene for normalization. 

### 3.8. Cream Formulation 

The cream manufacturing process has been elaborated with the inclusion of a list of materials in the supplementary material (Table S2). In brief, for this experiment, the cream was synthesized by combining materials from the aqueous and oil phases outlined in Table S2. Thorough mixing is conducted until complete homogeneity is achieved. Subsequently, each mixture underwent heating to a temperature exceeding 75 degrees, followed by the blending of the two phases for emulsification. Finally, the mixture was cooled to below 40 degrees, and HLCE, along with other essential ingredients, was then introduced for thorough mixing. This process, referred to as Addition I, represents the fundamental blending protocol employed in formulating the structure. The rationale for introducing the HLCE in Addition I is to prevent the degradation of active ingredients and mitigate any decline in activity. Thus, for this clinical trial, placebo and test creams were prepared with an identical composition. The sole distinction between these two creams lies in the presence or absence of the HLCE. Unlike in vitro experiments, in the course of the clinical trial, we added 1% HLCE to the cream following internal laboratory protocols, taking into account the skin penetration rate of the test substance.

### 3.9. Clinical Test 

To assess the efficacy of the HLCE in improving facial hyperpigmentation, 21 healthy Korean women aged 20–57 years with hyperpigmentation were enrolled. This study followed a double-blind, randomized, split-face trial design. Each participant applied the placebo cream to one side of their face and the HLCE cream to the other side, using an appropriate amount twice daily (morning and night) for 8 weeks. Clinical evaluations were conducted at four time points:0, 4, 6, and 8 weeks. This clinical study was approved by the Institutional Review Board of the P&K Skin Research Center (IRB No. P1702-89). Melanin levels in the skin were assessed using the melanin index, measured with a Mexameter ^®^ MX 18 (Courage + Khazaka electronic GmbH, Koln, Germany). Skin brightness was evaluated based on the L*value recorded using a CM-700d Spectrophotometer (Minolta, Osaka, Japan). All measurements were performed in triplicate. Visual assessments were performed using the Skin Color Evaluation Index (according to the Korean Food and Drug Administration) as follows (Table 2):

### 3.10. Statistical Analysis

Data are presented as the mean ± standard deviation derived from a minimum of three independent experiments. Statistical analysis was conducted using Student’s *t*-test, with only *p*-values less than 0.05 reported as statistically significant. The significance levels are indicated as follows: * *p* < 0.05, ** *p* < 0.01, and *** *p* < 0.001, indicating statistically significant differences compared to the control group. For the clinical tests, the visual assessment scores were analyzed using the Friedman test, followed by the Wilcoxon signed-rank test with Bonferroni correction. Other clinical assessment data were subjected to repeated-measures analysis of variance (ANOVA) followed by a paired *t*-test with Bonferroni correction. To assess differences between groups in visual assessment scores, the Mann–Whitney U test was applied for statistical significance analysis, whereas differences between groups in other clinical assessment data were evaluated using the independent *t*-test. In all instances, a normality test was initially applied, and differences were deemed statistically significant at *p* < 0.05. Statistical analyses were conducted using the SPSS software (version 19.0; IBM, Armonk, NY, USA).

## 4. Discussion

In general, it is significant that we found a new raw material, the HLCE, which may be applied in the field of cosmetics because it showed whitening effects, as proven by several in vitro tests and clinical trials. Despite the limited number of references, we inferred that the lotus has practical uses in various fields, including cosmetics and medicine, based on several theories. It is much easier to find a reference for the effects of the lotus or its byproducts in domestic journals or patents. In this study, we focused on the advantages of calluses, which specifically contain secondary metabolites. Notably, Zhu et al. presented a compilation of metabolites, including flavonoids and alkaloids, identified to date [30]. It is conceivable that certain chemicals mentioned in the report may be present in the HLCE. To maximize the characteristics of these callus, we obtained their extracts and conducted experiments by adding them to mammalian cells and cosmetics. Through our research, we confirmed that HLCE not only has the aforementioned effects but also plays a fundamental role in skin whitening, matching our hypothesis with the actual experimental results.

One of the key findings of this study was the extraction of a substance from the lotus ‘callus,’ for which there is a limited amount of literature available, especially regarding its effects on skin recovery or cosmetics. There are several studies on plant callus extracts, such as the antioxidant properties of *Stevia rebaudiana* callus using the 2,2-diphenyl-1-picrylhydrazyl assay [31], antidiabetic activity of *Aegle marmelos* in rabbits (in vivo) [19], anti-hepatotoxic effects of root callus extracts of *Cichorium intybus* L in rats (in vivo) [18], and analgesic effects of *Phyllanthus* tested in mice (in vivo) [32]. Although no research on lotus callus extract and its effect on the skin has been conducted, it is plausible that the callus and its extract contain some fundamental biocompounds according to the characteristics of the plant callus investigated in the past [33,34,35]. Therefore, we presume that the properties of lotus callus extracts are similar to those of plant extracts.

The lotus performs several functions. Yen et al. reported that lotus seed extracts have scavenging effects against reactive nitrogen species in vitro [36]. The seed extract was also reported to have antioxidant, antihemolytic, and nephroprotective properties when tested in experimental in vitro and in vivo models [37]. Moreover, lotus leaves have been used as a dietary herbal medicine in China [38]. Numerous studies have shown that the lotus has positive effects on skin whitening [39,40], which may be attributed to studies suggesting a relationship between biocompounds such as polyphenols and TYR activity [41]. Cuvelier et al. found that rosemary and sage extracts were significantly effective against oxidative stress. They identified several components, such as phenolic acids and flavonoids, in the extract using high-performance liquid chromatography [42]. Additionally, according to Azize, Jujube (Zizyphus lotus) is rich in polyphenols, vitamins, and minerals, which are effective against oxidative stress, inflammation, and the immune system [43]. These compounds like polyphenols play critical roles in melanogenesis [44]. Monnai et al. demonstrated that rice seeds enriched in polyphenols or flavonoids showed inhibitory effects by downregulating the mitogen-activated protein kinase signaling-mediated microphthalmia-associated transcription factor [45] and Allam et al. showed that polyphenols from *Vicia faba* L inhibit lipase activity and melanogenesis [46]. Moreover, it is suggested that polyphenol oxidase also plays a crucial role in melanin formation by promoting melanin synthesis [47]. Although the polyphenols mentioned above were not from the lotus, we presume that our results may have a germane relationship with these facts: polyphenol-like compounds from the HLCE promoted whitening effects and significantly inhibited melanin synthesis. 

Skin whitening has long been of interest and numerous products, food, and folk remedies are widely used for skin whitening [48,49]. However, as the focus on beauty continues to intensify, the formulation and development of cosmetics, among other products, has increasingly adopted more professional and scientific approaches. Many studies have shed light on the functionality of ingredients and the mechanisms underlying their application. Including raw materials such as polyphenols, we speculate that there may be several significant mechanisms or signaling pathways that mediate skin whitening via TYR suppression. Morikawa et al. suggested that alkaloids from the lotus (*Nelumbo nucifera*), such as nuciferine, nornuciferine, N-methylasimilobine, and pronuciferine, have strong correlations with melanogenesis inhibitory activity [41] and that these alkaloids become effective via the TYR inhibition mechanism [50]. Alkaloids from the lotus were found to intervene in and inhibit numerous signaling pathways, such as protein kinase C [51], Wnt/B-catenin [52], nuclear factor-kappa B [53], and phosphoinositide-3-kinase/Protein kinase B signaling pathways [54,55]. Fundamentally, these signaling pathways have been reported to be closely related to TYR signaling, which leads to the skin-whitening mechanism. Protein kinase C signaling has been suggested to activate TYR signaling [56,57], stimulating human melanogenesis [58]. Moreover, Wnt/B-catenin signaling activates melanogenesis [59], which is regulated by activated NF-kB signaling via Toll-like receptor 9 [60]. In addition, Chung et al. demonstrated that melanogenesis is inhibited by accelerating the phosphoinositide-3-kinase/AKT signaling pathway using an antimycotic agent [61]. The alkaloid components of *N. nucifera* have been reported to inhibit or activate many well-known signaling pathways, and these mechanisms have been shown to induce TYR suppression or inhibit melanogenesis. This evidence strongly supports the results related to the whitening effect of the HLCE.

Therefore, we suggest that the HLCE has the potential to prevent melanin synthesis and induce skin whitening. We did not develop any product using this material; however, we could at least manufacture a temporary cream for clinical testing. In general, the creams used for the clinical tests had a higher concentration of the target material than the concentration confirmed by the in vitro tests. This is generally due to official policies and numerous legislations in the cosmetics industry, as well as the outcomes of research [62,63]. The main reason for the higher dose was the rate of skin penetration of the targeted materials included in the products. Cells cultured in vitro usually grow within a single layer, whereas human skin has multiple layers of fats [64,65,66]. Most importantly, the results from our clinical trial revealed that melanin content was significantly reduced starting from the sixth week of cream administration. The brightness of the skin increased, followed by melanin reduction (Figure 5 and Figure 6). While additional experiments to analyze the components of the HLCE would have been highly beneficial, we regret noting that due to environmental constraints, we were unable to conduct such experiments. Nevertheless, one aspect remains unequivocal: within the HLCE, there are compounds with great promise that exhibit inhibitory effects on melanin synthesis. Therefore, it is imperative to elucidate the active constituents of the HLCE through further component analyses.

## 5. Conclusions

To the best of our knowledge, this is the first study to use lotus callus extract in cosmetics. The efficacy of the lotus has been elucidated in numerous reports to date, allowing us to reasonably speculate on the mechanisms by which the HLCE exerts its skin-whitening effects. In the future, it will be essential to directly analyze the components of the HLCE and confirm their association with the aforementioned mechanisms using sequencing or similar methods. However, to date, we have presented evidence of the skin-whitening effect of HLCE through in vitro and clinical trials, signifying a significant impact on skin whitening.

## Figures and Tables

**Table 1:** Composition of M4 for callus culture.

Medium Components	mg/L
NH_4_NO_3_	825
KNO_3_	950
MgSO_4_∙7H_2_O	185
MnSO_4_∙H_2_O	11.2
ZnSO_4_∙7H_2_O	4.3
CuSO_4_∙5H_2_O	0.013
KH_2_PO_4_	85
KI	0.42
CoCl_2_∙6H_2_O	0.013
CaCl_2_∙2H_2_O	220
H_3_BO_3_	3.1
Na_2_MoO_4_∙2H_2_O	0.13
FeSO_4_∙7H_2_O	13.93
Na_2_ –EDTA	18.63
Myo-inositol	6000
Thiamine HCl	0.4
2,4-Dichlorophenoxyacetic acid	0.3
6-benzyladenin	1
Sucrose	30,000
Gelrite	4000
pH adjusted to 5.8 Sterilized at 121℃ for 15 min	

**Table 2:** Intensity score table.

Bright & Clear		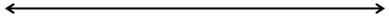						Dark & Dull	
1	2	3	4	5	6	7	8	9	10

## Data Availability

The data is contained within the article.

## Supplementary Materials

The following supporting information can be downloaded at: https://www.mdpi.com/article/10.3390/plants12233923/s1, Table S1: List of media used for callus induction; Table S2: Process and raw material list for cream manufacturing.
