# Supplementary material for: Skin-Whitening Effect of a Callus Extract of Nelumbo nucifera Isolate Haman
Source: Plants (Basel). 2023 Nov 21;12(23):3923. doi: 10.3390/plants12233923 (PMC10708243; doi:10.3390/plants12233923)
Supplement: Supplementary file 1 [file plants-12-03923-s001.zip › plants-2682272-supplementary.pdf]

Supplementary material

Table S1. Media used for callus induction of lotus from Haman region. All media were sterilized at 121°C, 15 min and pH was adjusted to 5.8.

	mg/L						
	M1	M2	M3	M5	M6	M7	M8
NH_4NO_3	-	1,650.0		825.0		1,650.0	
KNO_3	2,500.0	1,900.0	2,500.0	950.0	2,500.0	1,900.0	2,830.0
$MgSO_4 \cdot 7H_2O$	400.0	370.0	250.0	185.0	250.0	370.0	184.85
$(NH_4)_2SO_4$			134.0		134.0		462.49
$MnSO_4 \cdot H_2O$	10.0	22.3	10.0	11.2	10.0	22.3	2.98
$ZnSO_4 \cdot 7H_2O$	1.0	8.6	2.0	4.3	2.0	8.6	1.5
$CuSO_4 \cdot 5H_2O$	0.2	0.025	0.025	0.013	0.025	0.025	
KI	1.0	0.83	0.75	0.42	0.75	0.83	0.8
NaH_2PO_4			150.0		150.0		
KCl			300.0		300.0		
$CoCl_2 \cdot 6H_2O$	0.1	0.025	0.025	0.013	0.025	0.025	
$NH_2H_2PO_4$	300.0		150.0		150.0		
$CaCl_2 \cdot 2H_2O$	200.0		150.0	220.0	150.0	440.0	125.42
KH_2PO_4				85.0		170.0	313.0
H_3BO_3	5.0	6.2	3.0	3.1	3.0	6.2	1.6
$Na_2MoO_4 \cdot 2H_2O$	0.1	0.25	0.25	0.13	0.25	0.25	0.25
$FeSO_4 \cdot 7H_2O$	15.0	27.85	27.85	13.93	27.85	27.85	27.85
Na_2 –EDTA	20.0	37.25	37.25	18.63	28.0	37.25	37.25
Myo-inositol	1,000.0	100.0	100.0	6,000.0	100.0	100.0	100.0
Thiamine HCl	5.0	0.4	10.0	0.4	10.0	0.4	1.1
Nicotinic acid	5.0		1.0		1.0		0.5
Pyridoxine HCl	0.5		1.0		1.0		0.5
Sucrose	30,000.0	30,000.0	20,000.0	30,000.0	20,000.0	30,000.0	30,000.0
Phytagel	4,000.0	4,000.0	4,000.0	4,000.0	4,000.0	4,000.0	4,000.0
Proline							700.0
Glycine							2.0
Casein hydrolysate							100.0
2,4-Dichlorophenoxyacetic acid	0.5				1.5	1.0	
p-chlorophenoxyacetic acid	2.0						
Kinetin	0.1		0.1		0.1		
6-benzyladenin		1.0		1.0			
α-naphthaleneacetic acid		2.0	1.0		1.0		
Indole-3-acetic acid					0.1		

Table S2. Process and raw material list for cream manufacturing.

Phase	Component	Content (%)
Aqueous phase	Purified water	Up to 100
	Glycerin	10-25
	Betaine	-
	Sodium hyaluronate	-
	Thickener	q.s
	Chelating agent	q.s
Oil phase	PEG-100 stearate	0.1-2
	Glyceryl stearate	0.1-2
	Polysorbate 60	0.1-2
	Stearic acid	0.1-2
	Cetearyl alcohol	0.1-2
	Capric/caprylic triglyceride	10-30
	Tocopheryl acetate	0.1-0.5
Addition I	Fragrance	q.s
	Preservative	q.s
	Other additives	q.s

q.s: Quantum satis
